# Efficacy of 8% arginine associated with low-level laser therapy in the treatment of dentin hypersensitivity: randomized double-blind clinical trial

**DOI:** 10.1007/s10103-026-04927-z

**Published:** 2026-06-20

**Authors:** Viviane Leal Barbosa, Djuly Westenhofen, Paola Scherdien Silva, Roberto Zimmer, Harry Rivera Oballe, Sergio Augusto Quevedo Miguens Júnior, Eduardo Galia Reston

**Affiliations:** 1 Universidade Luterana do Brasil, Canoas, Brazil; 2 Pontifical Catholic University of Rio Grande do Sul, Porto Alegre, Brazil; 3 Universidade Federal de Pelotas, Pelotas, Brazil

**Keywords:** Dentin hypersensitivity, Arginine, Photobiomodulation, Low-level laser therapy, Randomized clinical trial

## Abstract

Purpose: To evaluate the efficacy of 8% arginine-based desensitizing toothpaste and photobiomodulation (PBM), used alone or in combination, in the treatment of dentin hypersensitivity (DH). Materials and methods: This randomized, double-blind, parallel-group clinical trial included participants with DH who were allocated into four groups: laser group (LG), arginine group (AG), combined laser and arginine group (LAG), and control group (CG). Clinical assessments were performed at baseline and after 2, 4, and 8 weeks. DH was evaluated using evaporative (air stimulus) and tactile tests applied to the cervical-buccal surface of the teeth, with pain intensity recorded using the Visual Analog Scale (VAS). Linear mixed-effects models were used for longitudinal continuous outcomes (Schiff and VAS scores), while generalized estimating equations were applied for tactile sensitivity outcomes, adopting a significance level of 5%. Results: A total of 60 participants were enrolled, and 54 completed the study. A significant reduction in DH was observed over time for both Schiff scores and VAS values (p < 0.001). Mean Schiff scores decreased from 5.0 (± 0.8) at baseline to 1.9 (± 1.3) at 8 weeks, while VAS scores also showed significant reductions. All groups demonstrated improvement in DH symptoms; however, no statistically significant differences were observed between interventions at any time point (p > 0.05). Conclusions: All evaluated therapies were effective in reducing DH over the 8-week follow-up period, with a progressive reduction in symptoms over time. However, no statistically significant differences were observed between the interventions, and no additive effect was identified for the combined therapy. Clinical trial registration: Brazilian Clinical Trials Registry ReBEC; RBR2vxzvkz, October 13^th^, 2023.

## Introduction

Dentin hypersensitivity (DH) is a common condition in clinical practice that negatively impacts patients’ quality of life [1, 2]. It is characterized by a short, sharp pain arising from exposed dentin in response to external stimuli—typically thermal, evaporative, tactile, or osmotic—that cannot be attributed to any other dental condition [3]. The prevalence of DH varies considerably across populations, with a pooled estimate of approximately 33.5% reported in a systematic review [1].

The hydrodynamic theory remains the most widely accepted explanation for DH, suggesting that fluid movement within dentinal tubules activates mechanosensitive nerve fibers, resulting in pain [4, 5]. Clinically, evaporative and tactile stimuli are commonly used to assess DH, and pain intensity is frequently measured using the Visual Analog Scale (VAS) [3, 6–8].

Several therapeutic approaches have been proposed, including agents that promote dentinal tubule occlusion and those that modulate neural transmission [9–11]. Among these, desensitizing toothpastes containing 8% arginine have demonstrated efficacy by forming a calcium-rich layer that occludes dentinal tubules [12, 13]. In parallel, photobiomodulation has been shown to reduce DH symptoms through modulation of inflammatory processes and neural activity [14–19].

Red (approximately 660 nm, within the red spectral range of ~ 600–700 nm) and near-infrared wavelengths (~ 808–980 nm) have been investigated in the management of DH, due to their potential effects on neural modulation and pain reduction [19–21]. In mineralized dental tissues, both wavelength ranges present low absorption by hydroxyapatite, allowing light transmission toward deeper structures, including the pulp.

Although both chemical and physical therapies have shown effectiveness, there is no consensus on a gold-standard treatment for DH, and the potential additive effect of combining therapies remains unclear [11, 22, 23]. Therefore, the aim of this randomized clinical trial was to evaluate the efficacy of 8% arginine-based desensitizing toothpaste and PBM, used alone or in combination, in the treatment of dentin hypersensitivity. The null hypothesis was that no difference would be observed between the interventions over time.

## Methodology

### Study design and ethical considerations

This study was designed as a randomized, double-blind, parallel-group clinical trial, conducted in accordance with the CONSORT guidelines. The protocol was approved by the Research Ethics Committee of the Lutheran University of Brazil (protocol n^o^. 5.326.784; CAAE: 30439120.7.0000.5349) and registered at the Brazilian Clinical Trials Registry (ReBEC – RBR-2vxzvkz). All participants provided written informed consent prior to enrollment.

### Eligibility criteria and recruitment

Participants were recruited from the dental clinics of the Lutheran University of Brazil between August 2022 and December 2023.

Individuals aged ≥ 18 years presenting with non-carious cervical lesions and dentin hypersensitivity (DH) in at least two non-adjacent teeth were included. DH was confirmed by an evaporative stimulus (air jet), defined as a score of 2 or 3 on the Schiff Cold Air Sensitivity Scale (0–3). Participants were periodontally healthy (probing depth ≤ 3, bleeding on probing < 10%, no clinical attachment loss ≥2 mm in at two non-adjacent interproximal sites, and visible plaque index (VPI) and gingival bleeding index (GBI) lower than 20%.

Exclusion criteria included allergy to the tested products, presence of oral mucosal lesions, pregnancy or breastfeeding, current chemotherapy and/or radiotherapy, individuals presenting endodontic lesions, extensive restorations, previous periodontal therapy, previous dental bleaching, current use of desensitizing toothpaste or participation in other clinical studies.

### Sample size calculation

Sample size was calculated based on a pilot study including 21 participants using G*Power software (version 3.1). Based on the mean and standard deviation of the Schiff score, an effect size of 0.53 was estimated, considering an alpha error of 5% and a power of 80%, resulting in a minimum required sample of 44 participants. To account for possible losses during follow-up, 60 participants were recruited.

### Randomization and blinding

Participants were randomly allocated into four groups using block randomization generated by Random.org (Randomness and Integrity Services Ltd., Dublin, Ireland) by an independent researcher not involved in other study phases.

Allocation concealment was ensured using sequentially numbered, opaque, sealed envelopes.

Both participants and the outcome assessor were blinded to group allocation. Toothpastes were removed from their original packaging and placed in identical opaque containers to maintain blinding.

### Interventions

All participants received standardized oral hygiene instructions, including toothbrushing twice daily, a soft-bristled toothbrush, and the toothpaste assigned to their respective groups for exclusive use throughout the study period. Groups LG and GC used a fluoridated toothpaste (1,450 ppm fluoride) and AG and LAG received and used a fluoridated toothpaste containing an additional desensitizing agent (8% arginine), as specified in the respective intervention to standardize oral conditions.

### Laser group (LG)

Participants received PBM using a Photon Lase III^®^ device (DMC^®^, São Paulo, Brazil), calibrated prior to the study according to the manufacturer’s specifications to ensure accurate power output.

The irradiation parameters were as follows: diodo laser, in a continuous mode, wavelength of 660 nm, output power of 100 mW (0.1 W), and spot size of 0.028 cm². The laser beam was applied perpendicular to the cervical-buccal surface of the tooth at a standardized distance of 0.5 cm, maintained using a metallic spacer coupled to the device.

Irradiation was performed at three points per tooth, with an exposure time of 28 s per point (total of 84 s per tooth). The total energy delivered was 8.4 J (power x irradiation time), corresponding to a nominal energy density of 300 J/cm², calculated based on the manufacturer-reported spot area of the device tip (0.028 cm^2^).

### Arginine group (AG)

Participants received a fluoridated toothpaste (1.14% sodium monofluorophosphate) containing 8% arginine as the active desensitizing agent and calcium carbonate (Colgate^®^ Sensitive Pro Relief Original™).

A standardized amount (~ 0.25 g, equivalent to a pea-sized portion) was applied topically to the cervical-buccal surface of the selected teeth using a gloved finger, for 1 min.

### Laser + arginine group (LAG)

Participants received the same procedures as described for the AG and LG groups. The intervention was performed sequentially, consisting of topical application of the arginine-based toothpaste followed immediately by PBM using the same irradiation parameters described above.

### Control group (CG)

Participants received simulated PBM (sham laser application), in which the device was activated with an audible signal but without emission of laser energy, along with a standard fluoridated toothpaste (1,450 ppm fluoride - Colgate^®^ Sensitive Maximum Cavity Protection™), without specific desensitizing agents.

### Outcome assessment

Assessments were performed at baseline and after 2, 4, and 8 weeks. Dentin hypersensitivity was evaluated using an evaporative stimulus test under relative isolation with cotton rolls. A standardized air jet from a triple syringe was applied perpendicular to the tooth surface at a distance of 1 cm, controlled by a polypropylene barrier. Sensitivity was recorded using the Schiff Cold Air Sensitivity Scale (0–3), with scores of 2 and 3 considered indicative of DH.

After a standardized interval of 3 min, a tactile test was performed using a #5 explorer probe, gently passed in a mesio-distal direction along the cervical-buccal surface. Tactile sensitivity was recorded as a binary variable (present/absent). Pain intensity was measured using a Visual Analog Scale (VAS), ranging from 0 (no pain) to 10 (worst pain imaginable), a validated method for pain assessment in DH studies [3]. The VAS was done before and immediately after the intervention at each evaluation time point (baseline, 2, 4 and 8 weeks).

### Statistical analysis

Descriptive statistics included absolute and relative frequencies for categorical variables and means ± standard deviations for continuous variables. Longitudinal changes in Schiff and VAS scores were analyzed using linear mixed-effects models, including fixed effects for group, time, and group × time interaction, and random intercepts for participants. Binary tactile sensitivity outcomes over time were analyzed using generalized estimating equations (GEE), considering within-subject correlation across repeated evaluations. All analyses were performed using R software (version 4.3.1), adopting a significance level of 5%.

## Results

### Participant characteristics

**Fig. 1:**
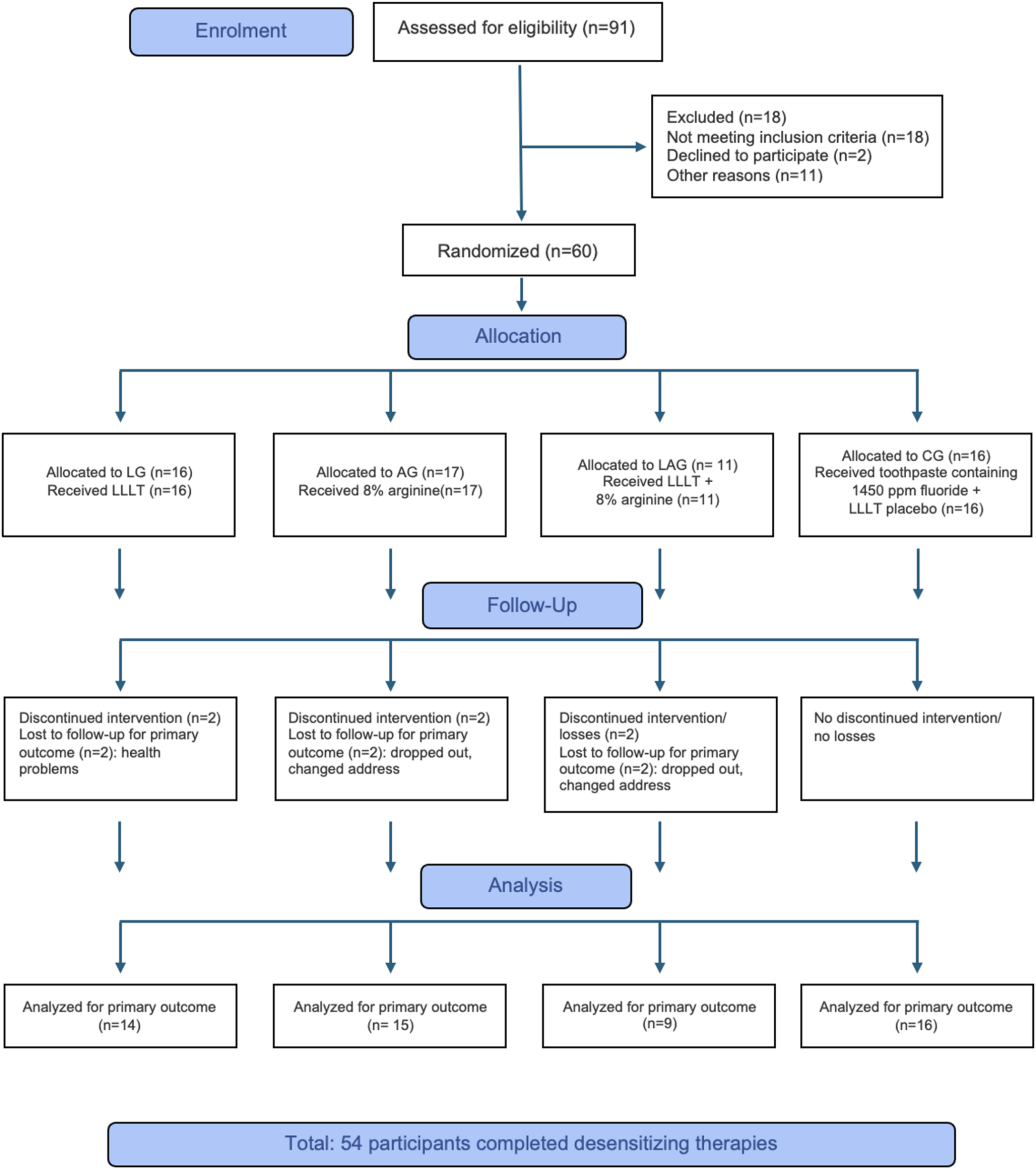
Flow diagram of participants throughout the study

A total of 60 participants were initially enrolled in the study. After losses to follow-up, 54 participants completed the 8-week evaluation period and were included in the final analysis (Fig. 1). A total of 120 teeth were evaluated, predominantly premolars (68.3%), followed by canines (18.0%), molars (14.2%), and incisors (2.5%), in both arches.

Flowchart of participant progress through the trial, including enrollment, randomization, allocation, follow-up, and analysis, in accordance with the CONSORT.

Participants had a mean age of 51.4 years (± 12.6), and most were female (76.6%). The majority were non-smokers (90%), without systemic comorbidities (61.7%), and reported frequent consumption of acidic foods such as citrus fruits (50%) and sugary foods (46.7%). No statistically significant differences in baseline characteristics were observed among the groups (Table 1).

**Table 1:** Baseline characteristics of participants according to intervention groups

**Variables**	Interventions-Groups					
	LG (*n* = 16)	AG (*n* = 17)	LAG (*n* = 11)	CG (*n* = 16)	Total (*n* = 60)	p*
Age (years)						
Mean (± SD)(Min-Max)	47.6 ± 12 (25–68)	51.1 ± 12.5 (31–68)	51.5 ± 15.4 (26–71)	55.2 ± 11.1 (32–70)	51.40 ± 12.6 (25–71)	0.407
Sex, n (%)						
Female	10 (62.5%)	14 (82.4%)	9 (81.8%)	13 (81.2%)	46 (76.6%)	0.518
Male	6 (37.5%)	3 (17.6%)	2 (18.2%)	3 (18.8%)	14 (23.3%)	
Skin color(self-reported), n (%)						
White	13 (81.2%)	16 (94.1%)	11 (100%)	15 (93.8%)	55 (91.7%)	0.602
Black	2 (12.5%)	1 (5.9%)	--	--	3 (5.0%)	
Brown	1 (6.2%)	--	--	1 (6.2%)	2 (3.3%)	
Comorbidities, n (%)						
Diabetes	1 (6.2%)	3 (17.6%)	--	2 (12.5%)	6 (10.0%)	0.577
Hypertension	4 (25.0%)	5 (29.4%)	2 (18.2%)	5 (31.2%)	16 (26.7%)	0.922
Others	2 (12.5%)	1 (5.9%)	1 (9.1%)	3 (18.8%)	7 (11.7%)	0.688
None	10 (62.5%)	11 (64.7%)	8 (72.7%)	8 (50.0%)	37 (61.7%)	0.668
Habits, n (%)						
Smoking						
Yes	1 (6.2%)	3 (17.6%)	1 (9.1%)	1 (6.2%)	6 (10.0%)	0.686
No	15 (93.8%)	14 (82.4%)	10 (90.9%)	15 (93.8%)	54 (90.0%)	
Dietary/habits, n (%)						
Sugary foods	9 (56.2%)	5 (29.4%)	5 (45.5%)	9 (56.2%)	28 (46.7%)	0.359
Soft drinks	6 (37.5%)	2 (11.8%)	--	4 (25.0%)	12 (20.0%)	0.077
Hot drinks	16 (100%)	14 (82.4%)	9 (81.8%)	13 (81.2%)	52 (86.7%)	0.377
Acidic foods	8 (50.0%)	9 (52.9%)	5 (45.5%)	8 (50.0%)	30 (50.0%)	0.985
Tooth type, n (%)						
Incisors	0 (0%)	0 (0%)	1 (4.5%)	2 (6.2%)	3 (2.5%)	0.846
Canine	5 (15.6%)	7 (20.6%)	2 (9.1%)	3 (9.4%)	17 (14.2%)	
Pre-molar	23 (71.9%)	21 (61.8%)	16 (72.7%)	23 (71.9%)	83 (69.2%)	
Molar	4 (12.5%)	6 (17.6%)	3 (13.6%)	4 (12.5%)	17 (14.2%)	

Legend: *LG* laser group, *AG* arginine group, *LAG* laser + arginine group, *CG* control group

Values are presented as mean ± standard deviation (SD) or absolute frequency (percentage)

*n *= absolute number of participants within each intervention group; % = relative frequency within each group

*p-values were obtained using one-way ANOVA for continuous variables and Pearson’s chi-square or Fisher’s exact test for categorical variables

### Overall effect over time

Considering all participants, there was a progressive and statistically significant reduction in dentin hypersensitivity over time. Mean Schiff scores decreased from 5.0 (± 0.8) at baseline to 1.9 (± 1.3) at 8 weeks (*p* < 0.001). Similarly, VAS scores showed a significant reduction over time for both evaporative and tactile stimuli (*p* < 0.001). These findings indicate a consistent improvement in DH symptoms across the follow-up period, regardless of the intervention (Table 2).

**Table 2:** Overall longitudinal changes in dentin hypersensitivity outcomes across evaluation periods, irrespective of intervention group

Variable	Baseline (*n* = 60)	2 weeks (*n* = 56)	4 weeks (*n* = 54)	8 weeks8 (*n* = 54)	Δ Value
SchiffScore	5.0 (± 0.8)^a^	3.5 (± 1.2)^b^	2.9 (± 1.2)^c^	1.9 (± 1.3)^d^	-3.1 (± 1.5)
VAS (pre-intervention assessment)	6.3 (± 2.1)^a^	3.4 (± 2.7)^b^	2.7 (± 2.6)^b^	1.8 (± 2.5)^c^	-3.5 (± 2.7)
VAS (post-intervention assessment)	5.1 (± 2.3)^a^	3.0 (± 2.4)^b^	2.2 (± 2.2)^b, c^	1.6(± 2.3)^c^	-0.7 (± 2.0)

Legend: Values are presented as mean ± standard deviation (SD)

Different superscript letters (a, b, c, d) indicate statistically significant differences between evaluation periods within the same variable according to Bonferroni post hoc comparisons (*p* < 0.05). Overall *p*-values for time effect were obtained from linear mixed-effects models

### Comparison between intervention groups

All intervention groups demonstrated reductions in Schiff and VAS scores over time (Table 2). However, no statistically significant differences were observed between groups at any evaluation period (*p* > 0.05). Linear mixed-effects models demonstrated a significant overall reduction in dentin hypersensitivity across follow-up evaluations (*p* < 0.001), with no significant differences between intervention groups or significant group × time interaction effects (*p* > 0.05).

These results indicate that all interventions were similarly effective over time, with no evidence of superiority of any specific treatment (Table 3).

**Table 3:** Longitudinal comparison of Schiff and VAS scores between intervention groups across evaluation periods

Variable	Intervention Groups				*p*
	LG	AG	LAG	CG	
Schiff Score					*p* ^*^
Baseline	4.8 ± 0.8^a^	5.1 ± 0.9^a^	5.1 ± 0.9^a^	5.1 ± 0.8^a^	0.573
2 weeks	3.6 ± 0.9^b^	3.7 ± 1.2^ab^	3.7 ± 1.9^b^	3.1 ± 0.9^b^	0.434
4 weeks	2.9 ± 0.9^b^	2.9 ± 1.0^b^	3.6 ± 1.8^bc^	2.6 ± 1.3^b^	0.356
8 weeks	1.9 ± 1.6^c^	2.1 ± 1.3^c^	2.0 ± 1.1^c^	1.6 ± 1.3^c^	0.695
Δ Value	-3.0 ± 1.7	-2.9 ± 1.5	-2.9 ± 0.9	-3.5 ± 1.7	0.686
VAS					*p* ^*^
Baseline					
Pre-intervention	6.2 ± 1.9ª	6.6 ± 2.0^a^	5.9 ± 2.3ª	6.3 ± 2.3ª	0.815
Post-intervention	5.5 ± 2.0^b^	5.4 ± 1.9^b^	4.9 ± 3.2^b^	4.5 ± 2.4^b^	0.666
2 weeks					
Pre-intervention	3.5 ± 2.5ª	3.5 ± 3.0^a^	3.4 ± 3.1ª	3.3 ± 2.5ª	0.995
Post-intervention	3.7 ± 2.0^a^	2.8 ± 2.4^b^	2.7 ± 3.1ª	2.7 ± 2.3ª	0.489
4 weeks					
Pre-intervention	3.1 ± 2.4ª	2.8 ± 2.8ª	3.5 ± 3.7ª	1.8 ± 1.7ª	0.649
Post-intervention	2.7 ± 2.1^a^	2.0 ± 1.9ª	3.6 ± 3.4ª	1.3 ± 1.2ª	0.219
8 weeks					
Pre-intervention	2.1 ± 2.8ª	2.0 ± 2.6ª	1.9 ± 3.4ª	1.1 ± 1.6ª	0.836
Post-intervention	2.0 ± 2.5^a^	1.8 ± 2.1ª	1.8 ± 3.1ª	0.8 ± 1.7ª	0.69
Δ Value	-3.7 ± 2.7	-3.7 ± 2.7	-3.4 ± 2.8	-3.1 ± 3.0	0.966

Legend: Values are presented as mean ± standard deviation (SD)

Different superscript letters (a, b, c) indicate statistically significant differences between evaluation periods within the same group according to Bonferroni post hoc comparisons (*p* < 0.05). Overall *p*-values for group, time, and group × time interaction effects were obtained from linear mixed-effects models

*ANOVA; **Kruskal-Wallis

### Graphical representation

**Fig. 2:**
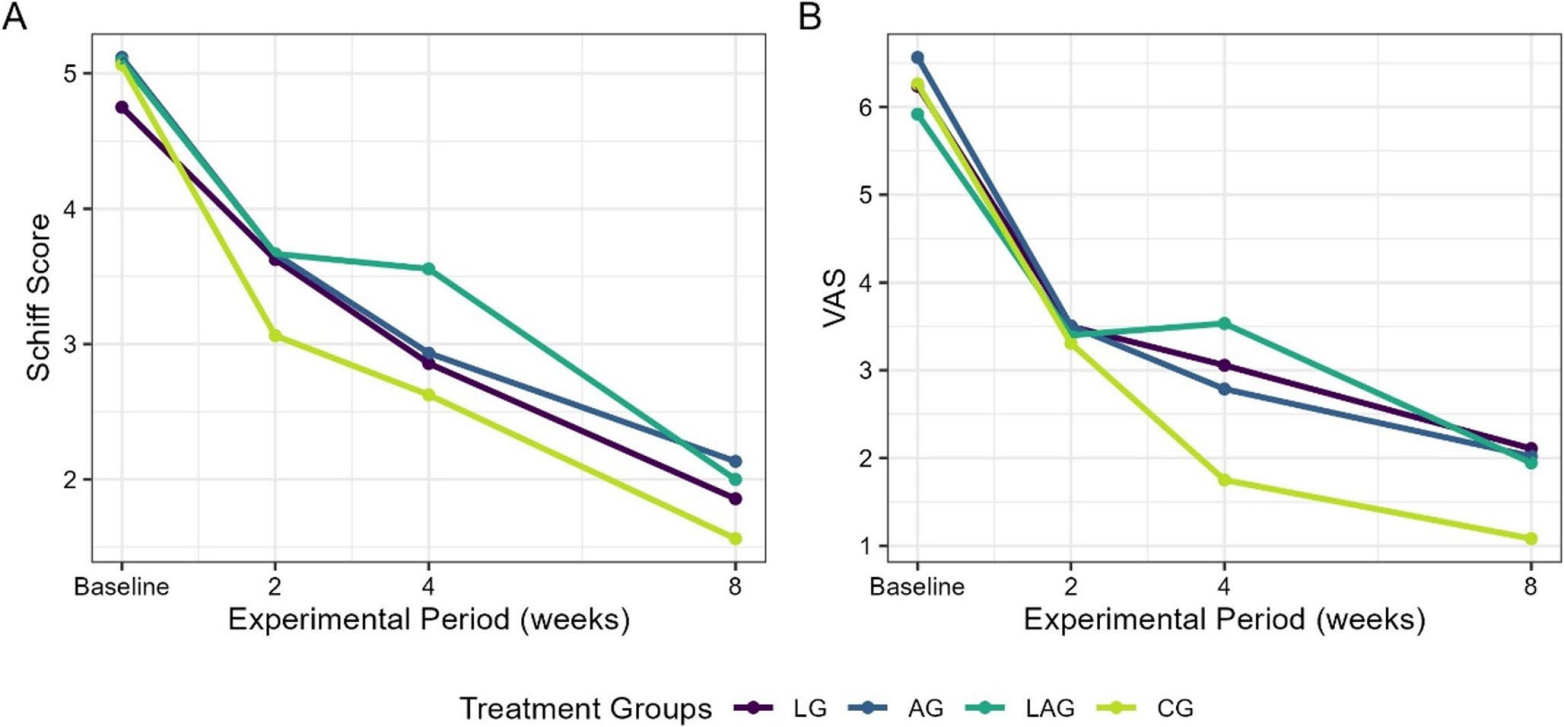
Changes in dentin hypersensitivity outcomes over time according to treatment groups. Legend: (**A**) Mean Schiff sensitivity scores over time. (**B**) Mean VAS scores over time. Data are presented as mean values at each time point (baseline, 2, 4, and 8 weeks). LG: Laser Group; AG: Arginine Group; LAG: Laser + Arginine Group; CG: Control Group

Figure 2 illustrates the longitudinal behavior of Schiff and VAS scores across the different groups.

A progressive reduction in scores is observed for all groups throughout the follow-up period, with similar trends and overlapping confidence intervals, reinforcing the absence of statistically significant differences between treatments.

### Association between tactile sensitivity and VAS

Table 4 presents the association between tactile sensitivity (recorded as present/absent) and VAS scores over time.

**Table 4:** Mean (± SD) VAS scores according to the presence or absence of tactile sensitivity across evaluation time points (baseline, 2, 4, and 8 weeks)

Time point	TactileSensitivity		
	Present	Absent	p*
Baseline	6.6 ± 2.5	5.1 ± 2.5	0.037
2 weeks	4.7 ± 2.7	3.2 ± 2.4	0.053
4 weeks	4.4 ± 2.8	2.4 ± 2.2	0.009
8 weeks	2.2 ± 2.5	1.7 ± 2.4	0.823

Legend: Values are presented as mean ± standard deviation (SD)

Comparisons were performed using generalized estimating equations (GEE) models to account for repeated measures over time

*p*-values represent comparisons between presence and absence of tactile sensitivity at each evaluation period

Higher mean VAS values were observed in teeth with tactile sensitivity compared to those without at baseline (*p* = 0.037) and at 4 weeks (*p* = 0.009). At 2 weeks, a borderline association was observed (*p* = 0.053), while no significant association was found at 8 weeks (*p* = 0.823).

The generalized estimating equations (GEE) model demonstrated that the presence of tactile sensitivity was associated with an average increase of approximately 1.46 VAS units during the early evaluation periods.

### Adverse effects

No adverse effects or complications related to the interventions were reported by participants during the follow-up period.

## Discussion

Several therapeutic approaches have been proposed for the management of dentin hypersensitivity (DH), primarily based on either the occlusion of dentinal tubules or the modulation of neural transmission [10, 11, 24]. However, DH is a multifactorial condition, and heterogeneity in study design, populations, follow-up duration, and intervention protocols limits direct comparisons across studies [10, 25, 26].

In the present randomized clinical trial, all interventions resulted in a statistically significant reduction in DH over time, regardless of the treatment group. This finding is consistent with previous studies reporting improvements in DH symptoms following both desensitizing agents and photobiomodulation therapy [8, 27, 28]. Importantly, no statistically significant differences were observed between groups at any evaluated time point, suggesting comparable effectiveness among the investigated therapeutic strategies.

The greater reduction observed during the initial follow-up period may reflect an early clinical response after treatment initiation and oral hygiene standardization, whereas the smaller variations at later evaluations suggest progressive stabilization of symptoms over time.

Photobiomodulation has been widely investigated in dentin hypersensitivity management, with evidence supporting analgesic and anti-inflammatory effects at different wavelengths, including red (approximately 660 nm, within the red spectral range of ~ 600–700 nm) and near-infrared wavelengths (~ 808–980 nm) [14, 15, 20, 28]. In the present study, reductions in pain levels were observed across all groups throughout follow-up. Within the PBM groups, the observed findings may be associated with the dosimetric parameters used (100 mW, 660 nm, 8.4 J, nominal energy density of 300 J/cm²) and with previously described mechanisms of photobiomodulation, including modulation of neural activity and inflammatory processes [20, 21].

Despite these findings, the absence of superior outcomes for PBM compared with the other groups is in agreement with systematic reviews reporting heterogeneous clinical effects of laser therapy in dentin hypersensitivity [17, 18, 23, 27, 29]. This variability may be attributed to differences in dosimetric parameters, wavelength selection, irradiation protocols, follow-up periods, and study populations, which may limit direct comparison between studies and contribute to heterogeneous clinical findings [29]. Therefore, greater methodological standardization in future randomized clinical trials may improve comparability of PBM outcomes in dentin hypersensitivity.

The use of an 8% arginine-based toothpaste also demonstrated a significant reduction in DH over time. Previous studies have shown that arginine-based formulations associated with calcium carbonate promote the formation of a mineral-rich layer that occludes dentinal tubules, thereby reducing dentinal fluid movement and sensitivity [9, 13]. The toothpaste evaluated in the present study contained 8% arginine associated with calcium carbonate, and the observed findings are consistent with this proposed mechanism [8, 12].

Notably, the combination of PBM and arginine did not result in an additive or synergistic effect compared with the isolated therapies. Although some studies have suggested a potential additive benefit [28, 30], the present findings do not support this hypothesis. This discrepancy may be explained by differences in study design, treatment protocols, and outcome assessment methodologies across studies [31].

An important finding was the reduction in DH observed in the control group. This effect may be partially explained by placebo responses and by behavioral changes associated with participants’ awareness of being monitored during the study period (Hawthorne effect), including greater attention to oral hygiene practices during follow-up [8].

Regarding pain assessment, higher VAS scores were observed following evaporative air stimulation compared with tactile stimulation. This finding may be related to the hydrodynamic changes induced by rapid air evaporation and fluid movement within exposed dentinal tubules [22]. In addition, the presence of tactile sensitivity was associated with higher VAS scores during the early follow-up periods, supporting the consistency between evaporative and tactile clinical assessments used for dentin hypersensitivity evaluation.

Some limitations should be considered when interpreting these findings. Although rigorous eligibility criteria and standardized protocols were adopted, dentin hypersensitivity is a multifactorial condition, and the specific etiology and duration of symptoms were not individually stratified among participants. In addition, participant losses during follow-up resulted in unbalanced final group sizes. Therefore, the absence of statistically significant differences between groups should be interpreted with caution.

Moreover, the specific etiology and duration of DH were not individually stratified among participants, rigorous eligibility and clinical screening criteria were adopted to minimize potential confounding factors. Individuals presenting conditions potentially affecting pain perception or dentin sensitivity, including periodontal disease, endodontic lesions, extensive restorations, previous periodontal therapy, dental bleaching, or current use of desensitizing agents, were excluded to ensure a clinically homogeneous sample.

Despite these limitations, the study adopted a randomized design with allocation concealment, blinded outcome assessment, and standardized intervention protocols to minimize potential sources of bias.

Within the limitations of this study, all evaluated interventions were associated with reductions in dentin hypersensitivity over time, with no evidence of superiority or additive effect among the tested therapies. Future randomized clinical trials, including factorial designs to better evaluate potential interaction effects between therapies, as well as larger samples and extended follow-up periods, are warranted to further investigate these findings.

## Conclusions

Considering the limitations of this study, all evaluated interventions were associated with reductions in dentin hypersensitivity throughout the follow-up period, with a more pronounced reduction during the initial evaluation interval followed by progressive stabilization over time. However, no statistically significant differences were observed between interventions at any evaluated time point, and no evidence of an additive effect was identified for the combined use of photobiomodulation and 8% arginine-based toothpaste.

## Data Availability

No datasets were generated or analysed during the current study.

## Declarations

### Clinical trial registration

Brazilian Clinical Trials Registry (ReBEC), identifier RBR-2vxzvkz, registered on October 13^th^, 2023.
